# Strategies to Reduce Tin and Other Metals in Electronic Cigarette Aerosol

**DOI:** 10.1371/journal.pone.0138933

**Published:** 2015-09-25

**Authors:** Monique Williams, An To, Krassimir Bozhilov, Prue Talbot

**Affiliations:** 1 Department of Cell Biology and Neuroscience, University of California Riverside, Riverside, California, United States of America; 2 Central Facility for Advanced Microscopy and Microanalysis, University of California Riverside, Riverside, California, United States of America; University of California, Merced, UNITED STATES

## Abstract

**Background:**

Metals are present in electronic cigarette (EC) fluid and aerosol and may present health risks to users.

**Objective:**

The objective of this study was to measure the amounts of tin, copper, zinc, silver, nickel and chromium in the aerosol from four brands of EC and to identify the sources of these metals by examining the elemental composition of the atomizer components.

**Methods:**

Four brands of popular EC were dissected and the cartomizers were examined microscopically. Elemental composition of cartomizer components was determined using integrated energy dispersive X-ray microanalysis, and the concentrations of the tin, copper, zinc silver, nickel, and chromium in the aerosol were determined for each brand using inductively coupled plasma optical emission spectroscopy.

**Results:**

All filaments were made of nickel and chromium. Thick wires were copper coated with either tin or silver. Wires were joined to each other by tin solder, brazing, or by brass clamps. High concentrations of tin were detected in the aerosol when tin solder joints were friable. Tin coating on copper wires also contributed to tin in the aerosol.

**Conclusions:**

Tin concentrations in EC aerosols varied both within and between brands. Tin in aerosol was reduced by coating the thick wire with silver rather than tin, placing stable tin solder joints outside the atomizing chamber, joining wires with brass clamps or by brazing rather than soldering wires. These data demonstrate the feasibility of removing tin and other unwanted metals from EC aerosol by altering designs and using materials of suitable quality.

## Introduction

Electronic cigarettes (EC), which are readily available on the Internet and in local convenient stores, have become a popular alternative to conventional cigarettes [1,2]. EC deliver aerosol to the user by heating a fluid containing nicotine, humectants, and flavorings [3,4]. The number of EC products is rapidly increasing with 460 brands of EC and over 7,000 unique flavors of refill fluid advertised on the Internet in January 2014 [5]. In several *in vitro* studies, cytotoxicity was associated with EC fluid and specific flavorings [6,7]. Various adverse health effects have been reported in online forums [8] and to the FDA [9]. Case reports have linked EC use with lipoid pneumonia [10] and eosinophilic pneumonitis [11]. However, EC are new products and relatively little is known about their beneficial or adverse health effects, and nothing is yet known about their long-term effects on health [12].

Over the past 9 years, several generations of EC have been introduced [13]. The first EC were cartridge style, which have a separate cartridge and atomizer. These evolved into the cartomizer style in which the atomizer and cartridge are combined into a single unit. More recently, larger tank models, which hold more fluid and usually have more powerful batteries, have been introduced. In all EC designs, there are metal components that could contribute particles and/or ions to the aerosol inhaled by users. Bystanders in the proximity of EC users could also be exposed to these metals. Long-term inhalation of metals can produce adverse health effects [14], which is of concern to EC users. Several studies have reported that metals, such as cadmium, nickel, lead, tin, zinc and copper are present in EC aerosol [15,16]. Some of the metals reported in EC are also present in cigarette smoke, but at lower concentrations [16]. Tin is of particular interest because it was found in milligram amounts in the fluid of one cartomizer brand [16]. Some of this tin was trapped in the Polyfil fibers of the cartomizer; however, both large (>500nm) and nanometer sized particles of tin were also present in the aerosol. Since EC performance is variable between and within brands [3,17,18], it is important to evaluate tin and other metals in fluids and aerosols of multiple brands and styles of EC.

The purposes of this study were to: (1) measure the amounts of tin, copper, zinc, silver, nickel, and chromium in the aerosol of four popular brands of EC, (2) determine which components in the cartomizers contribute metals to EC aerosols, and (3) determine which design features minimize introduction of metals into EC aerosol.

## Materials and Methods

### EC purchases

Four brands (A, B, C, and D) of EC were chosen for study. Each brand was popular at the time of purchase based on Google Internet searches using key words such as “electronic cigarettes”, e-cigs, ENDS, and “e-cigarettes”. EC were purchased from an Internet vendor (brands A, B, and C) or from a local drug store in Southern California (brand D). EC were purchased between 2012–2013 (brand A), between 2009–2012 (brand B), between 2011–2013 (brand C), and in 2014 (brand D). Brands A, B, and C are all cartomizer models, while brand D is a disposable device. We designate brand C as brand C (2011) or brand C (2013) to indicate the year it was purchased. Two of the three cartomizers from brand A will be presented separately and are designated A1 and A2.

### EC dissections

For each brand, three different units were carefully dissected to study their design features and to isolate parts for metal analysis.

For brand A, a saw was used to cut through the metal mouthpiece to expose the intact atomizing unit. Particles created during sawing were made of stainless steel, which was not analyzed in this study, and sawing was done away from the regions of analysis. The fibers were removed using forceps, exposing the sheath and wires. The air tube was soldered to the thick wires and was exposed once the mouthpiece and sheaths were removed.

For brand B, the mouthpiece and associated white plug and ring were first removed, allowing access to the inside of the EC. The outer Polyfil and inner fibers were removed using forceps, which exposed the air tube assembly and associated wires.

For brand C, the base of the air tube was pulled with pliers to separate it from the mouthpiece, which exposed the atomizer. The outer and inner fibers were unwrapped using forceps, exposing the air tube assembly and wires. For brand C, the products purchased in 2011 had the same name, external appearance, and packaging as those purchased in 2013, but upon dissection were found to differ in their design, as explained in the Results section.

For brand D, the terminal white plug was removed from the mouthpiece, which was then pulled straight off, exposing the Polyfil fibers that surround the atomizer. The outer and inner fibers were unwrapped using forceps, revealing the atomizer and its associated wires. The wires in this unit ran through the base of a plastic air tube and attached to the battery.

EC dissections were photographed using a Nikon SMZ 745 stereoscopic microscope and a Canon SLR digital camera.

### Aerosol preparation and analysis

Aerosol was generated using a smoking machine built at the University of Kentucky [3,19,20]. For each brand, aerosol solutions were prepared from three fresh cartomizers/disposable units and collected using a protocol described previously [16]. Aerosol was collected in 500 mL round bottom flasks covered with Parafilm submerged in an ice bath. Each 4.3 second puff of aerosol was allowed to fully dissolve in a solution of 10% nitric acid, 3% hydrochloric acid, and 87% deionized water before the next puff was added to the flask. 4.3 second puffs were collected as an earlier study had shown this to be the average puff duration for EC users [21]. A total of 60 puffs of aerosol were collected for each cartomizer, and room air samples were prepared in a similar manner. All samples were stored in 15 mL conical vials until analysis. An Optima 7300 PV (Perkin-Elmer, Waltham, MA) inductively coupled plasma optical emission spectrometer (ICP-OES) was used to quantify the concentrations of elements in each sample of aerosol and room air (ICP-OES analysis of metals S1 Material). The limit of quantification (LOQ) for each element is given in Table A in S1 Material along with additional data on the method. Room air values were subtracted from aerosol data to obtain final concentrations of each element in aerosols.

### Scanning electron microscopic analysis of dissected products

For each brand, dissected EC wires, the joints between the wires, the mouthpiece, air tube, and battery were mounted onto aluminum pin stubs covered with carbon tape. The morphology and elemental composition of each sample were analyzed using a FEI Co. NovaNano SEM 450 equipped with Oxford Instruments plc Aztec Synergy energy dispersive X-ray spectrometer (EDS) fitted with a X-Max50 50 mm^2^ SDD detector with energy resolution of 129 eV at MnKα in the Central Facility for Advanced Microscopy and Microanalysis at the University of California at Riverside. Scanning electron microscope (SEM) images were acquired in the secondary electron mode with a dedicated detector at 15 kV accelerating voltage of samples not coated with conductive film. The spatial distribution of chemical elements was determined by generating elemental EDS maps using Aztec software. The presence of minor elements below 1% weight in the analyzed components was determined by acquiring EDS spectra from selected points and quantifying the elemental concentrations.

## Results

### Elemental analysis of aerosol from each brand

**Fig 1:**
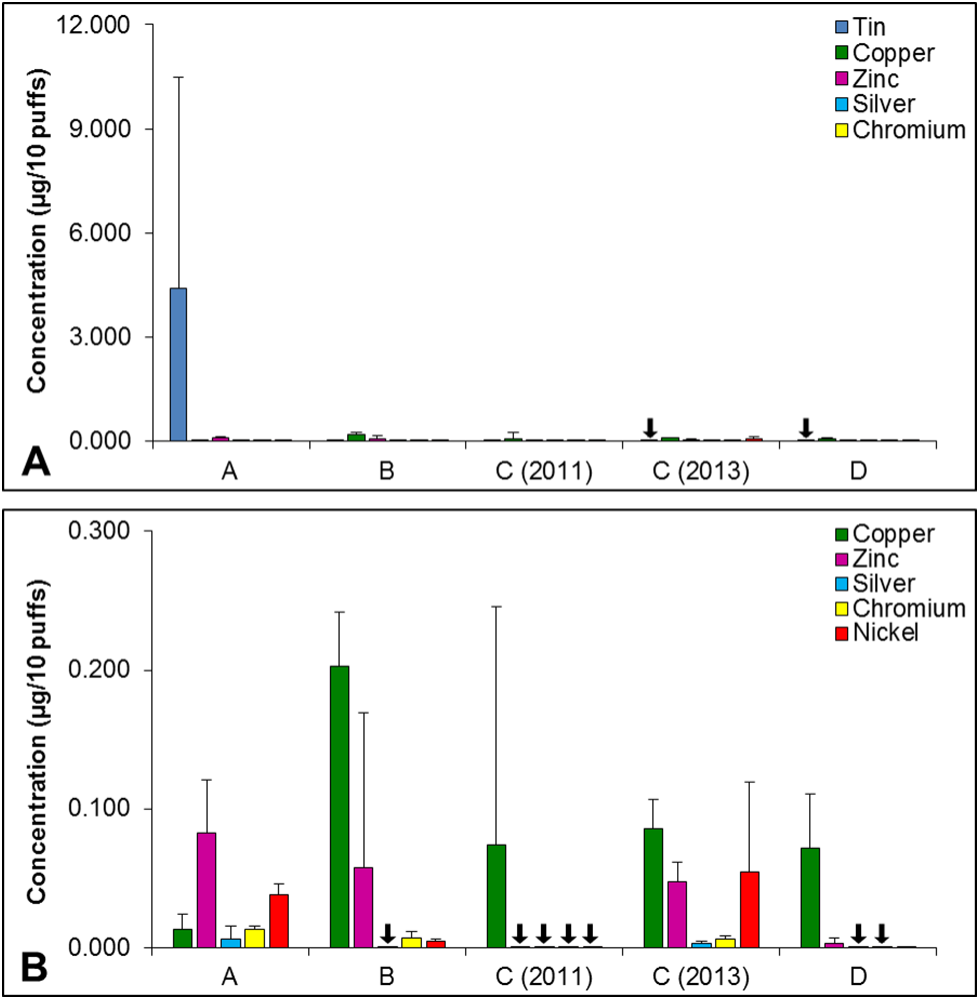
Total concentration of metals in aerosol of four brands of EC. (A) Total concentration of six metals (tin, copper, zinc, silver, chromium, nickel). (B) Total concentration of five metals (copper, zinc, silver, chromium, nickel). Each bar is the average ± standard deviation of three cartomizers. Black arrows indicate samples that were below the limit of quantification.

Tin, copper, zinc, silver, nickel and chromium concentrations were analyzed in the aerosol of three cartomizers from each of the four brands included in the study (Fig 1). Except for tin, concentrations were generally below 0.20 μg /10 puffs, and in some cases elements were not detectable, e.g., tin was not detected in the aerosol of brands C (2013) and D (Fig 1A). In one brand, C (2011), only copper and tin were detected, and in some cases there was significant variation in elemental concentration within a brand (e.g., copper in brand C (2011) and zinc in brand B had high variances) (Fig 1B). Silver concentrations were low or undetectable in all brands. Zinc concentrations ranged from below the level of quantification to 0.127 μg/10 puffs. Copper concentrations were higher in brands B-D than in brand A and were generally higher than zinc concentrations (Fig 1B). Chromium and nickel were either not detected, e.g. brand C (2011), or were detected at relatively low levels (e.g., brand D). Tin was the most variable element with brands B-D having relatively low concentrations of tin (range = 0 to 0.036 μg/10puffs), while brand A had on average 100 to 1000 times as much tin than the other three brands (Fig 1A). Within brand A, amounts of tin varied with the A1 cartomizer having a high concentration of 11.3 μg/10puffs and A2 having a low concentration of 0.398μg/10 puffs. Elemental analysis was next undertaken on individual atomizer components in each brand of EC to identify the source of each element.

### Elemental analysis of the components in brand A

**Fig 2:**
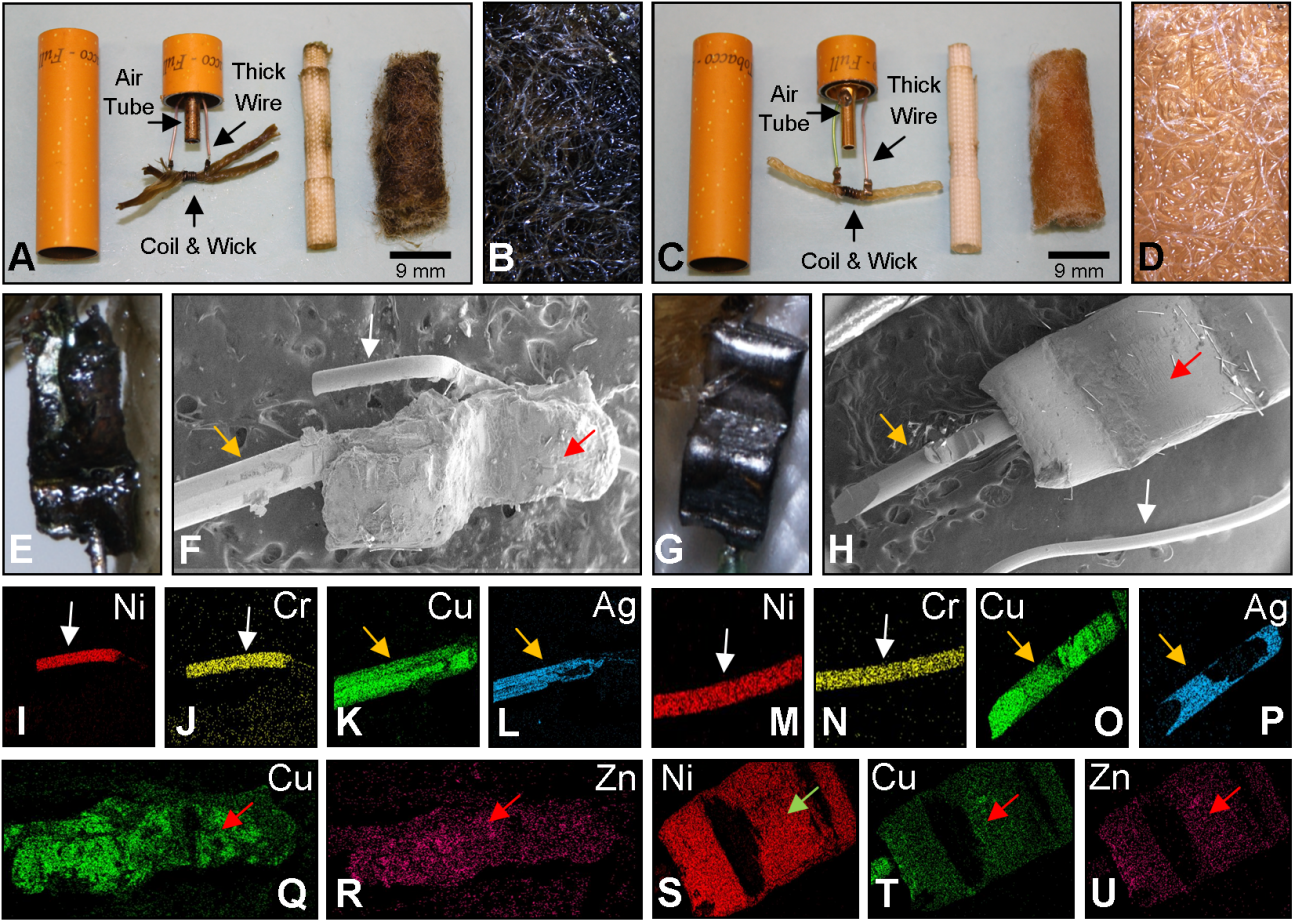
Cartomizer anatomy, fibers, and wire-to-wire joints in brand A. (A) Anatomy of a dissected cartomizer A1. (B) High magnification of the fibers from A1. (C) Anatomy of a dissected cartomizer A2. (D) High magnification of fibers from A2. (E) Micrograph of clamp joining wires in brand A1. (F) Scanning micrograph of clamp between thick wire and thin filament of A1 (2 mm). (G) Micrograph of clamp joining wires in cartomizer A2. (H) Scanning micrograph of clamp between thick wire and thin filament of A2 (2 mm).The thin wire (0.11 mm) from A1 was comprised of nickel (I) and chromium (J). The thick wire (0.25 mm) from A1 was comprised of copper (K) coated with silver (L). The thin wire (0.13 mm) from A2 was comprised of nickel (M) and chromium (N). Thick wire (0.25 mm) from A2 was comprised of copper (O) coated with silver (P). The clamp between the wires of A1 was comprised of mainly copper (Q) and zinc (R) (2 mm). The clamp between wires of A2 was nickel (S), copper (T), and zinc (U) (2 mm). White arrow = thin wire; Orange arrow = thick wire; Red arrow = the joints between the thick and thin wires; Green arrow = joint between wires.

**Fig 3:**
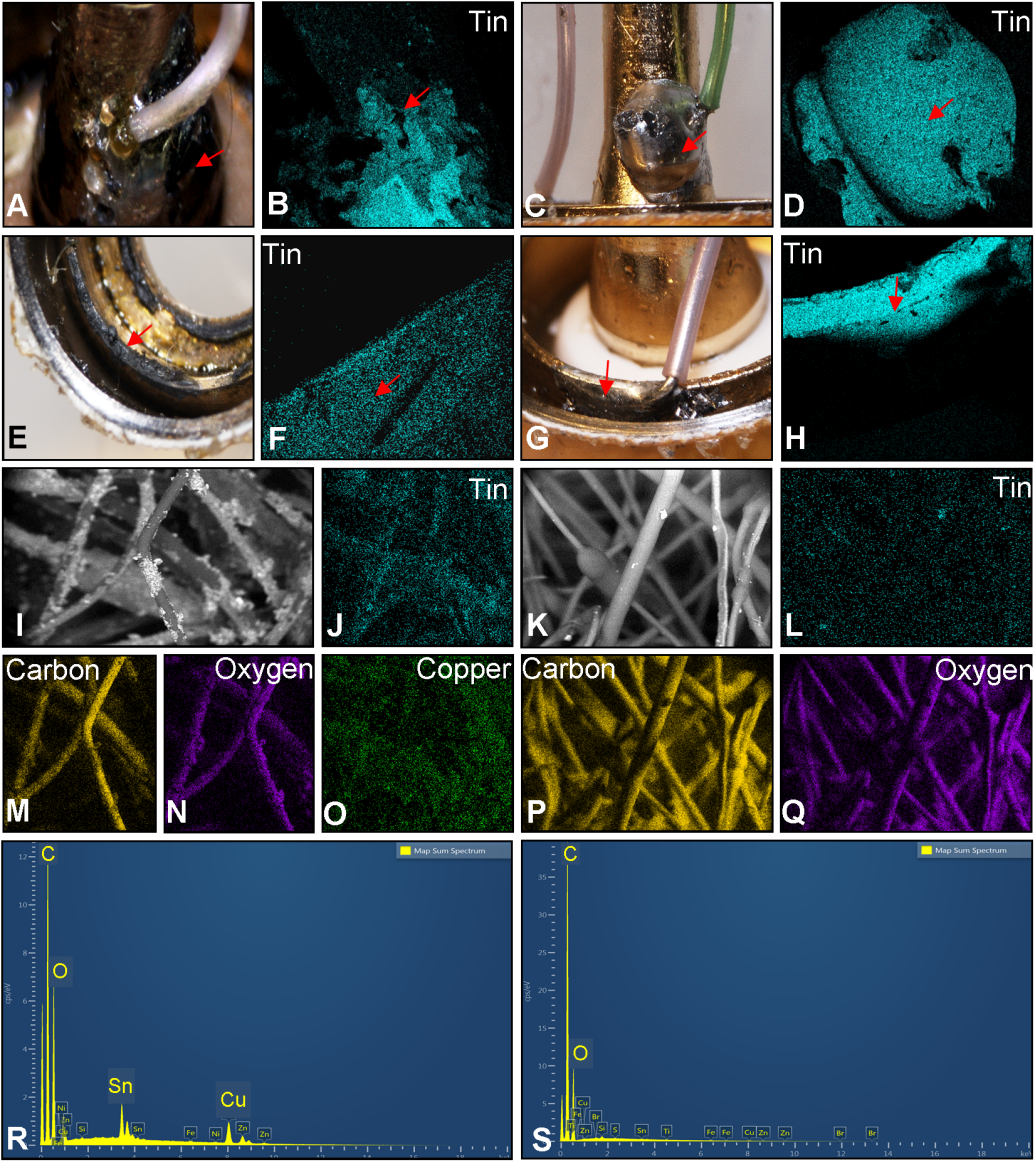
Joints between the thick wire and air tube or mouthpiece and Poly-fil fibers. (A) Micrograph of the solder joint between the thick wire and the air tube in A1. (B) Elemental map showing this solder joint in A1 is tin. (C) Micrograph of the solder joint between the thick wire and the air tube in A2. (D) Elemental map showing the solder joint in A2 is tin. (E) Micrograph of the solder joint between the thick wire and the mouthpiece in A1. (F) Elemental map showing the solder joint in A1 is tin. (G) Micrograph of the solder joint between the thick wire and the mouthpiece in A2. (H) Elemental map showing the solder joint in A2 is tin. (I) Electron micrograph of the fibers from A1. Elemental maps showing A1 fibers contains tin (J), carbon (M), oxygen (N), and copper (O). (K) Electron micrograph of the fibers of A2. Elemental maps showing the A2 fibers contains carbon (P) and oxygen (Q) but lack tin (L). Spectrum showing the fibers/fluid from A1 contains carbon, oxygen, tin, and copper (R), while the fibers/fluid from A2 contains carbon and oxygen (S). Red arrows = the joints between the thick wire and the air tube.

Because aerosol from brand A had high levels of tin, this brand was analyzed first to determine the source of the tin and why tin concentrations varied by approximately 30 fold between cartomizers from this brand. Cartomizers A1 and A2, which represented the high and low end of the range for tin production in brand A, were dissected and their anatomy and elemental composition were compared (Figs 2 and 3). Both cartomizers had thick and thin wires (filaments) which were joined at their ends to each other (Fig 2A and 2C). The opposite ends of the thick wires attached to the air-tube and wall of the mouthpiece. The coiled filament wrapped around a wick, and the entire assembly was surrounded by a fiberglass sheath, which was in turn was wrapped with Poly-fil. The major visible difference between the two cartomizers was the color of their fluids and Poly-fil fibers. The fluid/fibers from A1 were a dark brown in color (Fig 2A and 2B), whereas the fluid/fibers from A2 were light brown (Fig 2C and 2D).

Thick and thin wires were connected by a metal clamp (Fig 2E–2H). The clamp from A1 appeared rough and possibly damaged (Fig 2E and 2F), while the clamp from A2 was smooth-surfaced and appeared normal (Fig 2G and 2H). The thin wires in both the A1 and A2 cartomizers were made of nickel and chromium (Fig 2I, 2J, 2M and 2N), while the thick wire was copper coated with silver (Fig 2K, 2L, 2O and 2P). The clamps joining the two wires differed in that cartomizer A1 clamps were made mainly of copper with some zinc (Fig 2Q and 2R), while A2 was mainly nickel with some copper and zinc (Fig 2S–2U). The addition of a nickel coating to the clamp may have provided corrosion resistance, which could account for its better appearance.

The thick wire in brand A was attached to the air tube in both cartomizers via solder joints made of tin (Fig 3A–3D). The solder joint from A1 appeared friable and parts were missing (Fig 3A), whereas the solder joint from A2 was intact and shiny (Fig 3C). The other end of the thick wire was soldered to the wall of the mouthpiece nearest the battery-cartomizer interface (Fig 3E–3H). In the A1 cartomizer, this joint was largely missing (Fig 3E), and upon complete dissection, the thick wire had detached from this joint. The residue of solder that remained on the mouthpiece in A1 was predominantly comprised of tin (Fig 3F). In cartomizer A2, the solder joint to the mouthpiece was intact (Fig 3G), and this solder was also made of tin (Fig 3H).

The fibers in A1 were comprised of carbon and oxygen and were coated with tin and copper (Fig 3I and 3J, 3M–3O and 3R). In contrast, the fibers from A2 contained only carbon and oxygen (Fig 3K, 3L, 3P, 3Q and 3S). The above data are consistent with the idea that tin in the aerosol of brand A came from tin solder joints that were unstable and that stability of solder joints varied between brand A cartomizers.

### Design and elemental analysis of brands B, C, and D

**Fig 4:**
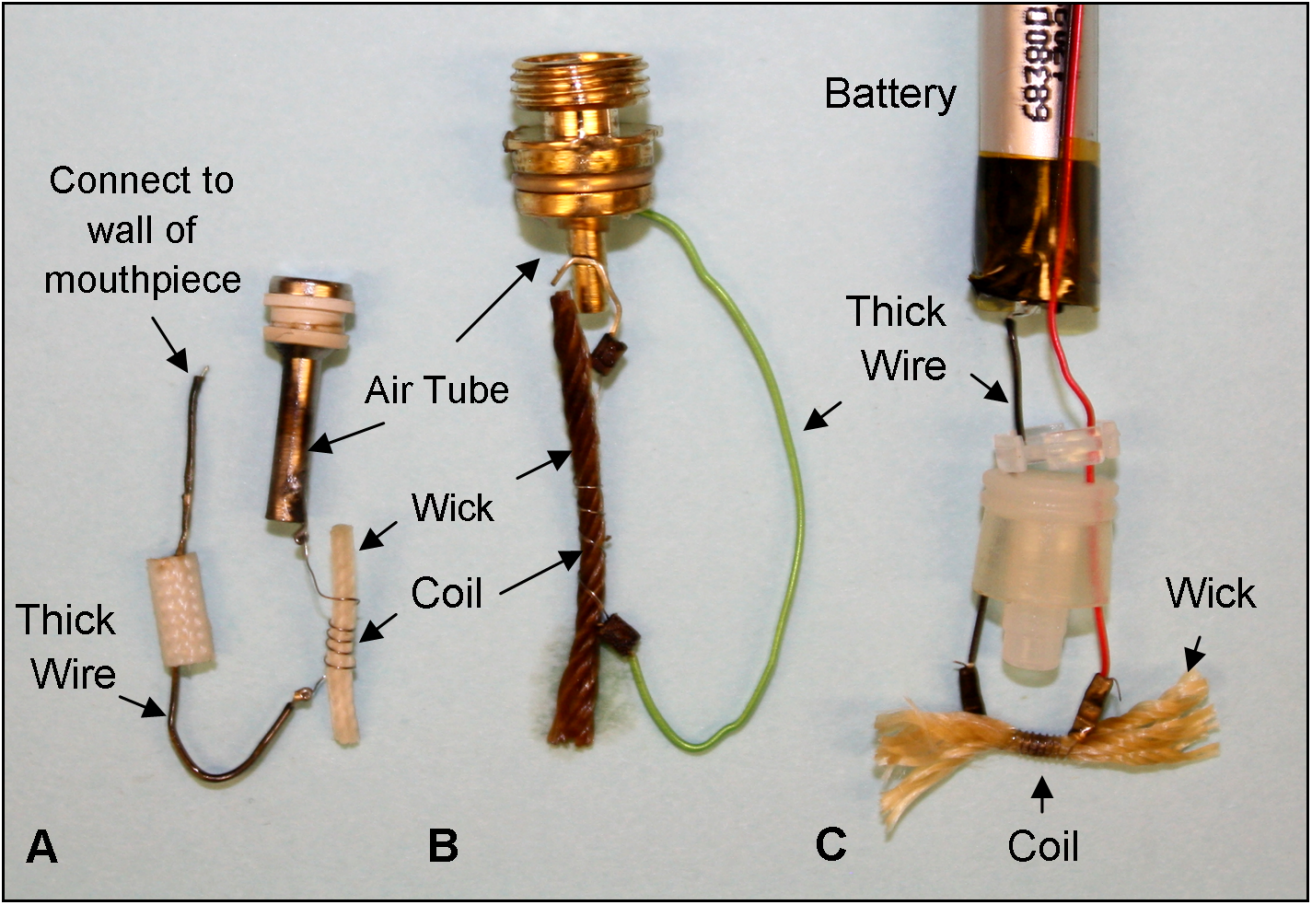
Anatomy of dissected EC atomizers for Brands B, C (2013), and D. The coiled thin wire (filament), wick, thick wire, air tube and battery are shown for the three brands.

The other three brands were dissected and analyzed using SEM-EDS to better understand how design features and manufacturing materials affected the occurrence of metals in EC aerosols. The basic design of the cartomizers in brands B, C and D was similar to that of brand A (Fig 4). Each had a thin wire or coiled filament that wrapped around a wick. The filament was connected to a thicker wire that in turn connected to: (1) the air tube and wall of the mouthpiece, in brand B; (2) the air tube and the metal at the base of the air tube in brand C; and (3) the battery which lies outside of the cartomizer compartment in brand D.

### Elemental composition of the wires in brands B, C, and D

**Fig 5:**
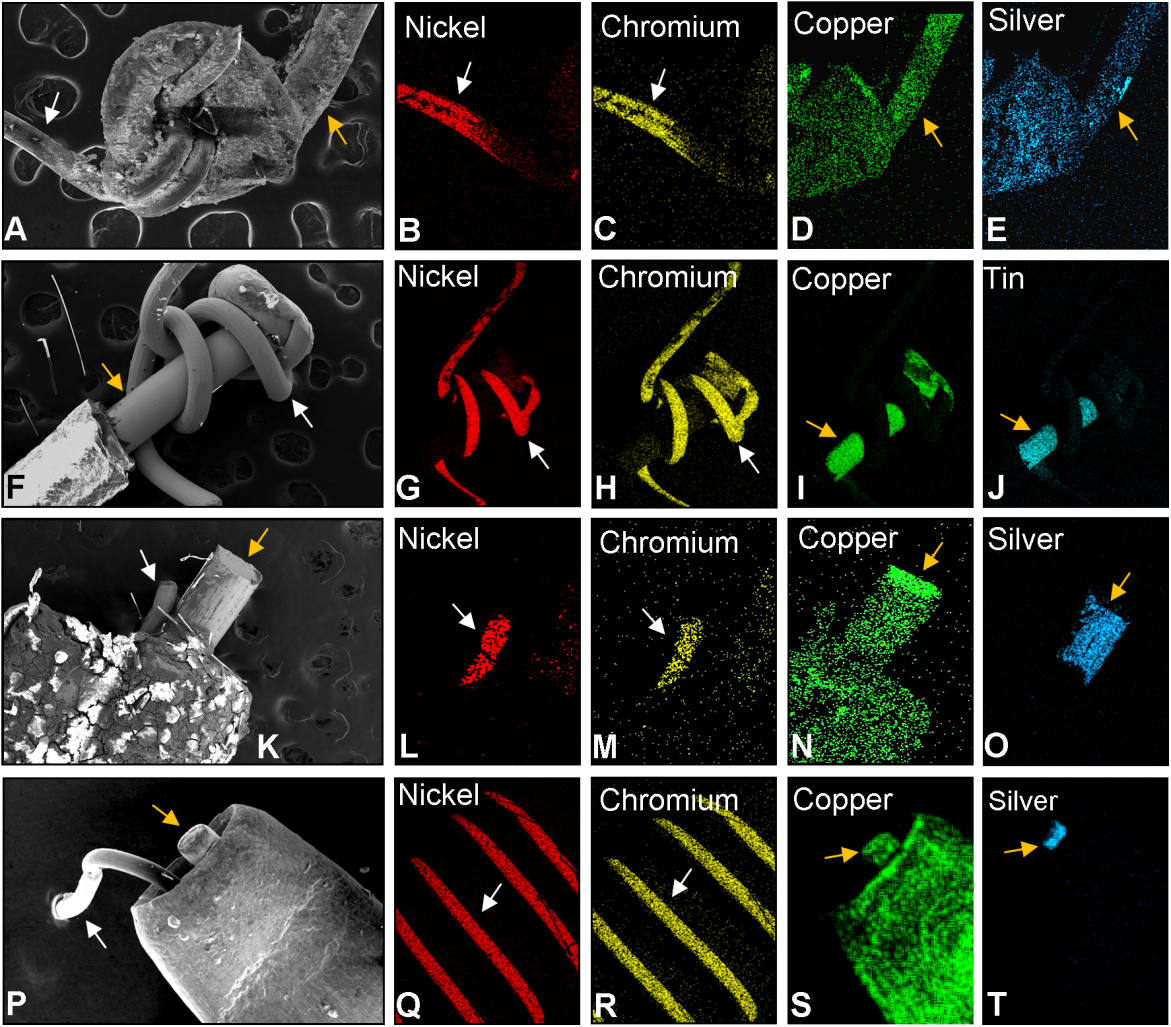
Elemental Analysis of the EC wires. (A) Scanning electron micrograph (SEM) of brand B solder joint. (B-C) Thin filament (0.15 mm) from brand B containing nickel and chromium. (D-E) Thick wire (0.33 mm) from brand B containing copper coated with silver. (F) SEM of brand C (2011) showing brazed joint between thin and thick wires. (G-H) Thin wire (0.14 mm) from brand C (2011) containing nickel and chromium. (I-J) Thick wire (0.25 mm) from brand C (2011) containing copper coated with tin. (K) SEM of brand C (2013) wires joined by clamp. (L-M) Thin wire (0.14 mm) from brand C (2013) containing nickel and chromium. (N-O) Thick wire (0.35 mm) from brand C (2013) containing copper coated with silver. (P) SEM of brand D showing brass clamp surrounding the thin and thick wires. (Q-R) Thin wire (0.13 mm) coiled around the wick from brand D showing nickel and chromium. (S-T) Thick wire (0.27 mm) from brand D containing copper coated with silver. White arrow = thin wire; Orange arrow = thick wire.

In brand B, the thin wire coiled around a loop of the thick wire (Fig 5A). The thin wire was made of a nickel-chromium alloy (Fig 5B and 5C), while the thick wire was made of copper coated with silver (Fig 5D and 5E).

In brand C (2011), most of the thick wire was coated with plastic, and at the joint between wires, the thin wire wrapped around the end of the thick wire (Fig 5F). The thin wire was comprised of a nickel-chromium alloy (Fig 5G and 5H), as seen in brand B; however, unlike brand B, the copper wire in brand C (2011) was coated with tin (Fig 5I and 5J). In brand C (2013) (Fig 5K), the thin and thick wires were crimped together inside a metal casing and were made of nickel and chromium (Fig 5L and 5M), and the thick copper wire was coated with silver (Fig 5N and 5O).

In brand D, the thick and thin wires were crimped together inside a metal casing or clamp (Fig 5P). The thin wire was again made of a nickel-chromium alloy (Fig 5Q and 5R), and the thick wire was copper coated with silver (Fig 5S and 5T).

### Elemental composition of the joints between wires

**Fig 6:**
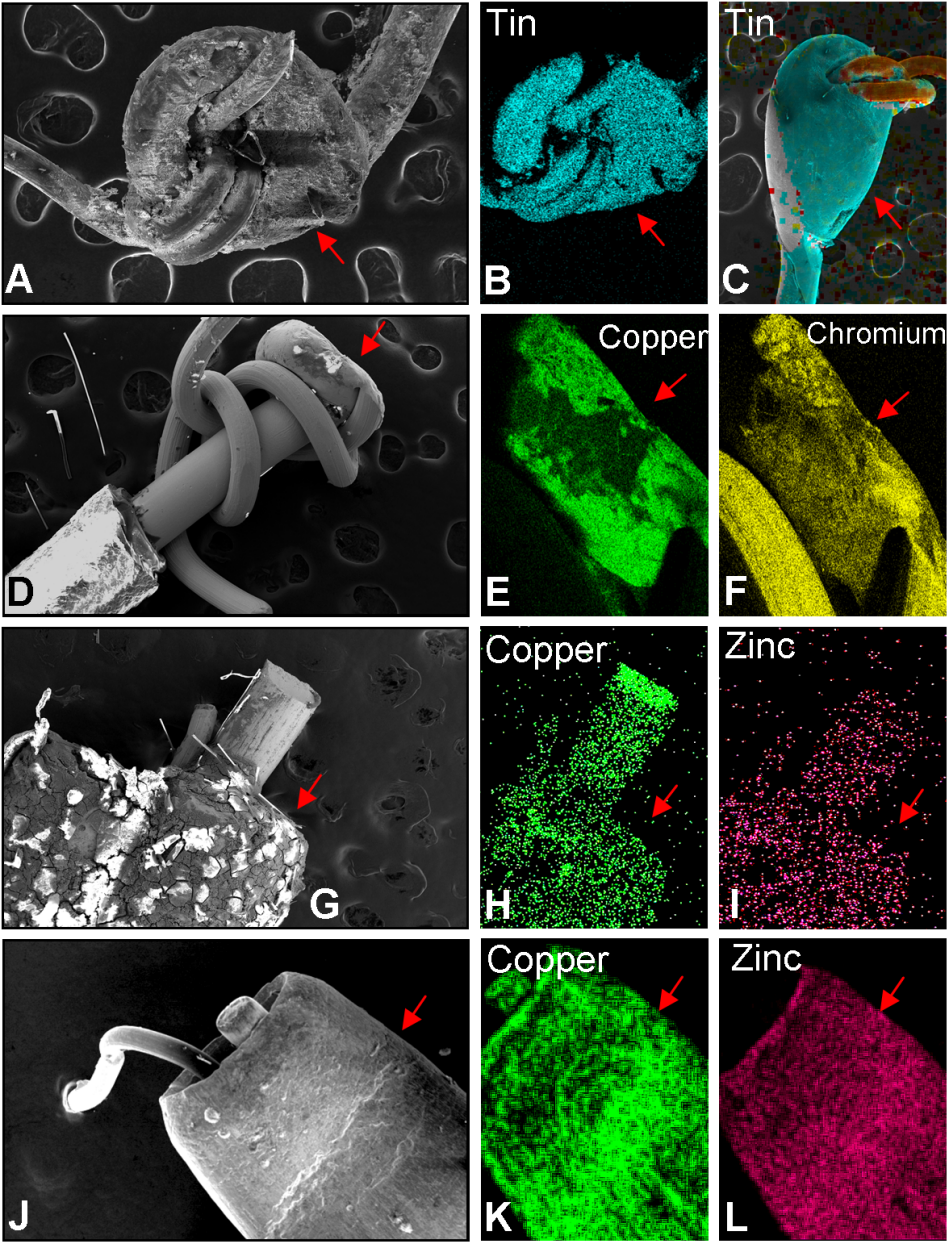
Elemental Analysis of the joints between wires in EC. (A) SEM of poor quality solder joint from brand B. (B) Elemental map showing brand B solder joints (1.0 mm) were made of tin. (C) SEM and elemental map showing a better quality solder joint from brand B. (D) SEM of brand C (2011) showing brazed wires (0.75 mm). (E-F) The wire joint from brand C (2011) showing copper and chromium in the brazed area. (G) SEM of the clamp used in brand C (2013) (1.65 mm). (H-I) Clamp joining wires in brand C (2013) is made of copper and zinc. (J) SEM of joined wires in brand D (1.41 mm). (K-L) Clamp in brand D is made of copper and zinc. Red arrow = joint between thick and thin wires.

The joints between the thick and thin wires in brand B (Fig 6A) were comprised of tin solder (Fig 6B and 6C). The majority of solder joints in brand B were of poor quality, and portions of the solder were missing (Fig 6A and 6B), while occasionally solder joints were intact (Fig 6C). Lead was not detected in the solder joints.

In brand C (2011), the joints between the wound wires consisted mainly of copper and chromium, which appeared to be brazed together (Fig 6D–6F). In the model purchased in 2013, the wires were joined together by a small clamp comprised of copper-zinc alloy, which is characteristic of brass (Fig 6G–6I).

In brand D, the two wires were also joined by a copper-zinc alloy or brass clamp (Fig 6J–6L).

### Joints between wires and the air tube or battery

**Fig 7:**
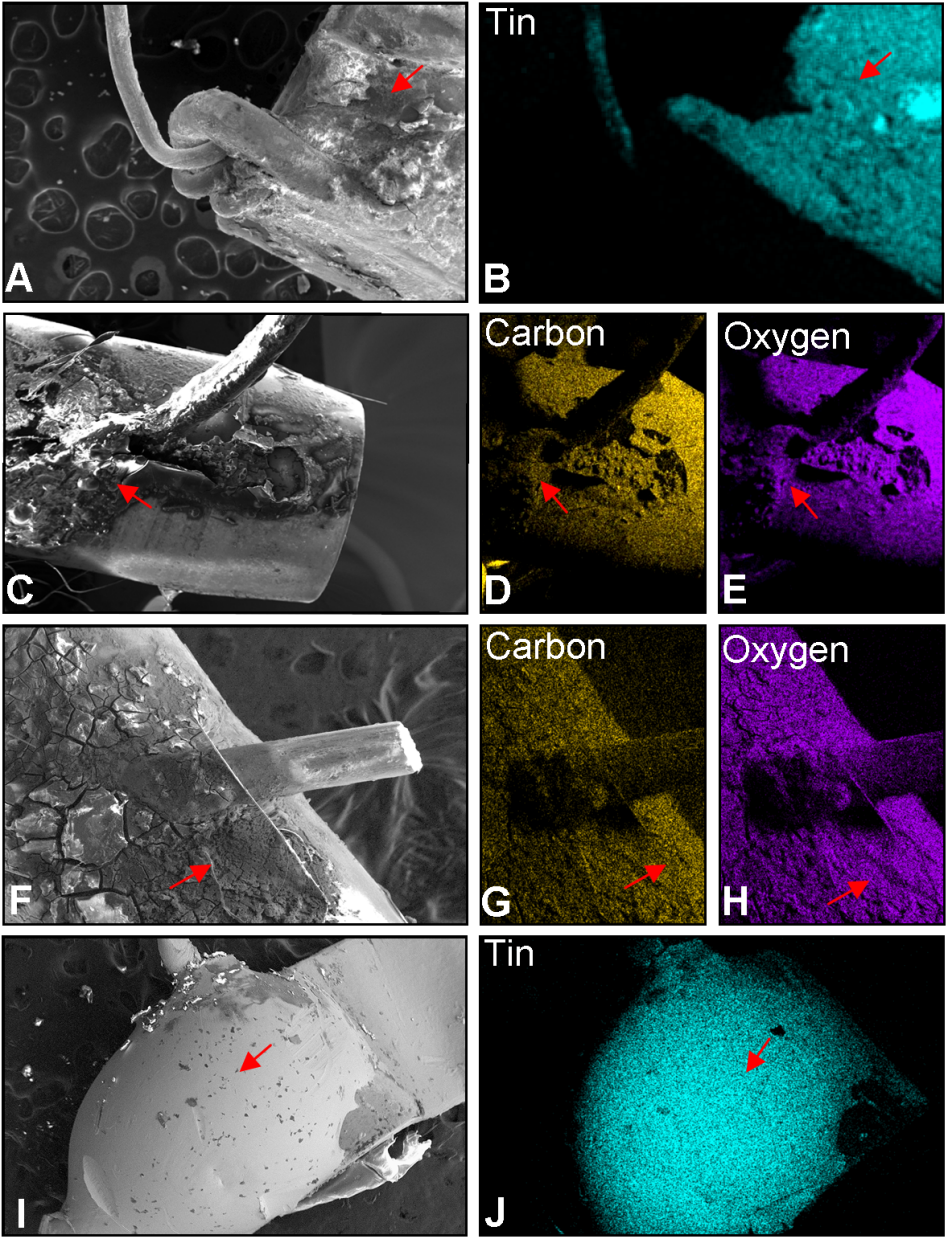
Elemental analysis of joints between the EC wire and air tube or EC wire and battery. (A) SEM of joint between thick wire and air tube of brand B (2 mm). (B) Elemental map showing joint contains tin. (C) SEM of joint between thick wire and air tube in Brand C (2011) (2 mm). (D-E) Elemental maps showing this joint contains carbon and oxygen. (F) SEM of joint between thick wire and air tube for brand C (2013) (2 mm). (G-H) Elemental map of the joint contains carbon and oxygen. (I) SEM of the joint between thick wire and the battery (1.71 mm) for brand D. (J). Elemental map showing this joint is comprised of tin. Red arrowheads = the joints between the thick wire and the air tube or battery.

The two thick wires were joined to the air tube or battery by different mechanisms. In cartomizers from brand B, the thick wire was joined to the air tube and wall of the mouthpiece by tin solder joints (Fig 7A and 7B). The solder joints in B did not contain lead. Solder quality in brand B was poor and solder joints were often fractured and partly missing.

In both the 2011 and 2013 versions of brand C (Fig 7C and 7F), the thick wire was attached to the air tube and base of the air tube by a brown substance that appeared with the stereoscopic microscope to be glue (not shown). The brown substance contained carbon and oxygen (Fig 7D, 7E, 7G and 7H), consistent with it being an organic compound or glue.

In brand D, the thick wires passed through the base of a plastic air tube and were attached directly to the battery by tin solder joints, which did not contain lead (Fig 7I and 7J). One solder joint was at the end of the battery nearest the cartomizer chamber, while the other solder joint was near the LED at the tip of the battery. Morphologically, solder joints in brand D were all of good quality.

## Discussion

The long term cumulative health effects of inhaling mixtures of metals in EC aerosol are not known, but it would be advantageous to remove metals from EC aerosols if possible. This study focused on tin, copper, zinc, silver, chromium and nickel in EC aerosol and identified the likely sources of each metal by examining the composition of the components in the atomizing chamber (Table 1). In addition, EC design features that minimized the concentration of these elements in aerosols were determined by comparing the concentrations of metals in the aerosol to the quality and composition of metal parts in the atomizing chamber. The amounts of these elements in aerosols varied significantly between brands, and for some elements, the variations were high within brands (e.g. tin in brand A, zinc in brand B, and copper in brand C (2011)), supporting the idea that quality control in manufacture of EC is not rigorous.

**Table 1:** Summary of Elements in EC Wires and Joints.

Brand	Thin Filament	Thick Wire	Wire toWire Joint	Wire to Air Tube/Mouthpiece/Battery Joint
A	Nickel, Chromium	Copper, Silver Coated	Zinc, Copper Clamp	Tin Solder
A1	Nickel, Chromium	Copper, Silver Coated	Zinc, Copper Clamp	Tin Solder
A2	Nickel, Chromium	Copper, Silver Coated	Nickel, Zinc, Copper Clamp	Tin Solder
B	Nickel, Chromium	Copper, Silver Coated	Tin Solder	Tin Solder
C (2011)	Nickel, Chromium	Copper, Tin Coated	Chromium and Copper Braze	Organic Glue
C (2013)	Nickel, Chromium	Copper, Silver Coated	Zinc and Copper Clamp	Organic Glue
D	Nickel, Chromium	Copper, Silver Coated	Zinc and Copper Clamp	Tin Solder

Silver was not abundant in aerosol from any of the four brands, but interestingly was highest (0.018 μg/10 puffs) in the A2 cartomizer, while undetectable in the A1 counterpart. This again shows how data can vary significantly between cartomizers within one brand. Silver in the aerosol most likely came from the silver coating on the thick copper wire. Silver was used to coat this wire in all products expect brand C, which had a tin coated copper wire, and no silver was detected in the aerosol of brand C. The thick wire was coated with plastic, and this coating may have reduced the amount of silver getting into the aerosol.

Zinc concentrations in aerosol ranged from below the limit of quantification (brand C (2011) to a high of 0.127 μg/10 puffs (cartomizer A1). Zinc was not detected in components used to manufacture brand C (2011), which also lacked zinc in its aerosol. Wires in brand C (2011) were joined by brazing, not by a brass clamp. Interestingly, zinc was detected in the aerosol of brand B even though no zinc was found in the cartomizer components that were analyzed.

Copper was present in all aerosols except those from cartomizer A1. Amounts ranged from below the limit of quantification to 0.203 μg/10 puffs. The likely sources of copper in aerosols were the thick copper wire, which was present in all products, and the copper-containing clamps that joined the thick and thin wires in all products except brand B.

Tin concentration in aerosols varied significantly among the four brands. Tin was absent from the aerosol of brand D but present at over 11 μg/10 puffs in cartomizer A1. Even within brands, the concentrations of tin in aerosols were variable (e.g., cartomizer A1 vs cartomizer A2). The tin in the aerosol of cartomizer A1 appeared to come from faulty solder joints between the air-tube and thick wire and between the mouthpiece and thick wire. The A2 cartomizer had relatively intact solder joints and lower levels of tin in its aerosol than A1, which would explain the within brand variation. Although the A2 cartomizer had less tin in its aerosol than A1, A2 produced aerosol with at least 10 times as much tin as brands B, C, and D. These differences in the tin concentrations in aerosols from brand A and brands C and D were due mainly to differences in the presence and/or stability of the tin solder joints. However, the differences between brands A and B were caused by another factor. Brand B had been studied previously and was reported to have friable solder joints and mg amounts of tin in its cartomizer fluid [16]. Interestingly brand B had much less tin in its aerosol than brand A. This was because most tin particles in brand B were trapped on the inner dense fibers that surrounded the wires [16].These fibers, which were not present in brand A, filtered out much of the tin in brand B before it could enter the Poly-fil and pass into the aerosol. In brand A, Poly-fil alone did not provide an effective filter, and tin readily passed through the Poly-fil into the aerosol.

Tin solder joints were not used in brands C (2011) and C (2013), which is consistent with them having low levels of tin in their aerosols. Brand C (2011), which had no tin solder joints, did have some tin in its aerosol, which probably came from the tin coating on the copper wire. This was the only brand to coat the thick wire with tin.

Lead, which was formerly included in tin to stabilize solder joints, was not detected in the solder of any of the brands studied, consistent with China’s prohibition on using lead in tin solder [22]. This may have made the solder joints in some brands less stable. During the rapid temperature cycling that occurs when EC are puffed, the tin solder could melt and be distributed throughout the cartomizer fluid, fibers, and aerosol. It is not clear why some of the solder joints were more stable than others, but tin has a relatively low melting temperature (231.9°C), which may be exceeded in some EC products, leading to degradation of the solder joints.

In brand D, the battery and atomizing chambers are not strictly isolated from each other, and during use air flows through the battery chamber into the atomizing chamber. Tin solder joints were used to attach the thick wires to the battery and LED at the tip of the EC. While these joints did not have lead, they appeared stable and tin in the aerosol was reduced to non-detectable levels, probably because the solder was located outside of the atomizing chamber, where the temperature would be lower during puffing.

The concentration of tin in EC aerosol can be reduced by joining wires with alternative methods that do not involve solder. Brand C (2011) had wires that wrapped around each other and appeared to be brazed, not soldered. In brands C (2013) and D, the wires were joined together by brass clamps. The concentrations of tin in the aerosol of both versions of brand C and brand D were lower than in brand B. Tin was also found in the aerosol of brand C (2013), however, the origin of this tin is unknown.

Replacing wire-to-wire tin solder joints (brand B) with brass clamps (all other brands) did not increase the levels of copper in the aerosols and only slightly increased the level of zinc in brand A. This may be because brass is inherently more stable than tin solder, particularly under atomizing conditions.

Although the use of clamps in place of tin solder in wire joints reduced tin in the aerosol, the products that used such clamps did not always produce aerosol reliably. In a study conducted by our lab last year [23], human participants found that brand C (2013) was not easy to use, and because it did not produce much aerosol, it was eventually discontinued in the study. The poor performance of brand C (2013) may be related to the method of joining wire; however other factors may also have caused poor performance. For brand D, 5 of 11 units were not capable of producing aerosol when used on the smoking machine. This could be because contact between the wires in the clamp was faulty in some units. If this is the source of the problem, it could be overcome with better clamping methods.

A limitation of this study is the inability of ICP-OES to distinguish large particles, which would contribute most of the mass to the values in Fig 1, from nanoparticles which would likely be negligible with ICP-OES. Our previous study showed that brand B aerosol contained tin nanoparticles as well as larger tin particles [16]. While brand D aerosol did not have measureable amounts of tin, tin ions or nanoparticles may have been present, but below the level of quantification by ICP-OES. The contribution of tin nanoparticles and tin ions to EC should be studied further in the future. Also these data may underestimate element concentrations as aerosols were generated using the lowest air flow rate that produced a robust puff of aerosol. There are no standards for EC puffing and many users take large puff volumes [23] and may be exposed to significantly higher concentrations of metals than reported here. Finally, factors in aerosol preparation, such as complete capture of metal nanoparticles, may influence concentrations. However, nanoparticles probably contribute little to the overall mass of each metal, and all samples were prepared in the same manner making relative comparison among samples valid.

In summary, quality control in the manufacturing and labeling of EC has been a concern since their introduction [24,17,25,26]. In this study, the levels of tin in EC aerosols were variable between and within brands, demonstrating a lack of quality control in the manufacture of these products. Poor quality solder joints correlated with higher levels of tin in EC aerosols. These data support the idea that tin in EC could be reduced to negligible levels by: (1) improving the quality of tin solder joints in EC, (2) replacing tin solder with brass clamps, and/or (3) moving high quality tin solder joints outside the atomizing unit, as was done in brand D. Lowering levels of metals in EC aerosols would decrease the possibility of adverse health effects developing with prolonged use.

## Supporting Information

S1 Material Additional Materials and Methods and Limit of Quantifications Table. (DOCX)
