# Supplementary material for: Strategies to Reduce Tin and Other Metals in Electronic Cigarette Aerosol
Source: PLoS One. 2015 Sep 25;10(9):e0138933. doi: 10.1371/journal.pone.0138933 (PMC4583845; doi:10.1371/journal.pone.0138933)
Supplement: S1 Material — (DOCX) [file pone.0138933.s001.docx]

**Supplementary Materials and Methods**

**ICP-OES Analysis of Metals**

Water and reagents: For ICP-OES, Milli-Q System water (Millipore, USA) at 18.5 mOhm of resistance was used to make the nitric acid solutions for dissolving metals in aerosols. The nitric acid was purchased from Macron Chemicals (Avanter Performance Materials, Inc, Center Valley, PA) and was AR Select (ACS) for Trace Element Analysis. The hydrochloric acid was purchased from Fisher Scientific (Fair Lawn, NJ) and was Certified ACS PLUS. Examination of samples with a NanoSight instrument (Malvern, England) revealed that no particles or nanoparticles were present in the solutions containing aerosol after 48 hours of incubation in nitric acid/water.

Analysis of metals: An ICP OES Perkin –Elmer Optima 7300 DV (Perkin Elmer USA) with an autosampler, a Perkin-Elmer Nebulizer (N0777036 REV A, Cyclonic spray chamber Optima 5300DV, Quartz 7mm baffle drain line) and a segmented array detector charge coupled device detector (SCD) was used for all analyses. The ICP-OES was calibrated daily using Perkin-Elmer Multi-element calibration standards Plus #2, #3, #4, and #5. Quality control checks on calibration were then run using NIST standard reference materials by Ultra Scientific (trace metal sample, catalogue number QCI-700A North Kingstown, RI). Running conditions were plasma flow = 15 L/min, auxiliary flow 0.2 L/min, nebulizer flow of 0.75 L/min, radio frequency power 1450 W, sample flow rate= 0.80 mL/min, and a read delay time of 12 sec. Yttrium at 2.5 ppm was run in line with sample introduction into the nebulizer and used as an internal standard. The blank contained distilled deionized water with 1% nitric acid. Each sample was run in triplicate. When interference was observed for any element, additional peaks were monitored to identify the best wavelength for quantification. The concentrations of each element in the blank were subtracted from the measured concentrations in each sample. Samples of room air made the same way as the EC aerosol samples were run with each batch of samples, and room air values were subtracted from measured concentrations of each element in the aerosols.

| **Supplemental Table A: Limits of Quantification** | |
| --- | --- |
| Analyte Name | mg/L |
| Ag | 0.002 |
| Al | 0.004 |
| As | 0.008 |
| B | 0.003 |
| Ba | 0.000 |
| Bi | 0.011 |
| Ca | 0.003 |
| Cd | 0.001 |
| Cr | 0.002 |
| Co | 0.001 |
| Cu | 0.002 |
| Fe | 0.001 |
| Ge | 0.009 |
| In | 0.007 |
| Ir | 0.242 |
| K | 0.002 |
| La | 0.001 |
| Mg | 0.000 |
| Mn | 0.000 |
| Mo | 0.002 |
| Na | 0.001 |
| Ni | 0.001 |
| Pb | 0.007 |
| Pd | 0.008 |
| Rb | 0.004 |
| Se | 0.017 |
| Si | 0.003 |
| Sn | 0.004 |
| Sr | 0.000 |
| Ti | 0.000 |
| V | 0.001 |
| W | 0.003 |
| Zn | 0.001 |
| Zr | 0.001 |
